# The impact of Shanghai’s comprehensive smoke-free legislation on hospitalization and mortality rates of ischemic heart disease: An interrupted time series analysis

**DOI:** 10.18332/tid/207350

**Published:** 2025-09-24

**Authors:** Lihang Sun, Huiting Yu, De Chen, Dan Qin, Ying Shi, Yafei Hu, Jingrong Gao, Chenchen Xie, Xin Chen, Haiyin Wang

**Affiliations:** 1 Shanghai Health Development Research Center, Shanghai, China; 2 Shanghai Municipal Center for Disease Control and Prevention, Shanghai, China; 3 Shanghai Municipal Center for Health Promotion, Shanghai, China; 4 School of Pharmaceutical Sciences and Yunnan Provincial Key Laboratory of Pharmacology for Natural Products, Kunming Medical University, Kunming, China

**Keywords:** public health, tobacco-related disease, tobacco control, smoking/harm reduction, interrupted time series

## Abstract

**INTRODUCTION:**

Smoking and secondhand smoke are major global health threats, significantly contributing to the burden of ischemic heart disease (IHD). Despite the implementation of tobacco control policies worldwide, limited evidence exists on the health impacts in Shanghai. This study evaluates the effects of Shanghai’s 2017 smoke-free legislation on IHD hospitalization and mortality rates.

**METHODS:**

We conducted Interrupted Time Series (ITS) method to analyze monthly data on IHD hospitalizations and mortality among registered residents of Shanghai from July 2013 to December 2021. Age-standardized rate, Poisson and negative binomial regression models were performed to control for covariates.

**RESULTS:**

We included 898535 hospitalizations and 180658 deaths caused by IHD from July 2013 to December 2021 in Shanghai. Following policy implementation, there was a significant immediate increase (β=8.29; 95% CI: 2.45–14.13) and post-trend decline (β= -0.73; 95% CI: -0.93 – -0.54) in IHD hospitalization, which is estimated to have prevented approximately 890 hospitalizations per year. Subgroup analysis revealed that the long-term decrease was more pronounced in individuals aged ≥65 years (β= -1.72; 95% CI: -2.21 – -1.24), compared to those aged 35–64 years (β= -0.33; 95% CI: -0.42 – -0.25). However, mortality rates showed no statistically significant immediate (β= -0.90, 95% CI: -4.76–2.95) or long-term changes (β= -0.0075; 95% CI: -0.14–0.12).

**CONCLUSIONS:**

Shanghai’s comprehensive smoke-free legislation appears to be associated with a significant long-term reduction in hospitalization rates and a modest decrease in mortality rates from ischemic heart disease, particularly among older adults. These findings support the potential cardiovascular health benefits of smoke-free policies, which provide useful evidence for other cities considering the adoption or reinforcement of comprehensive public smoking bans to help reduce the burden of cardiovascular disease and improve population health.

## INTRODUCTION

Smoking remains one of the leading preventable causes of death worldwide. According to the World Health Organization (WHO), more than 8 million people die each year from smoking-related diseases, including approximately 1.3 million non-smokers who are exposed to secondhand smoke, highlighting its substantial harm to cardiovascular health, particularly ischemic heart disease (IHD)^1^. IHD is one of the most common and severe smoking-related diseases and has remained the leading cause of death globally over the past two decades^2^. Recent studies indicate that smokers face a two to three times higher risk of developing heart disease^3^.

To address the health threat posed by tobacco use, 67 countries have adopted comprehensive smoke-free laws banning smoking in indoor public places and workplaces^4^. Evidence from international studies suggests that such policies are associated with significant reductions in cardiovascular morbidity and mortality. For instance, after Ireland implemented a national smoke-free law in 2004, mortality from IHD declined by 26%^5^. Similar effects were observed in Chile, where comprehensive indoor smoking bans significantly reduced hospitalizations and deaths from IHD, preventing around 4758 deaths from 2013 to 2017^6^.

China is the world’s largest consumer of tobacco products, currently facing major challenges in tobacco control. With over 300 million smokers and high smoking prevalence among men (50.5%), tobaccorelated diseases cause more than 1 million deaths annually, and this number is projected to reach 3 million by 2050 without effective interventions^7,8^. To address the tobacco-related health crisis, China ratified the WHO Framework Convention on Tobacco Control (FCTC) in 2006^9^. Since then, over 20 cities have implemented smoke-free policies, with some reporting health benefits consistent with international evidence. For example, tobacco control interventions led to an annual reduction of 16% in acute myocardial infarction mortality among individuals aged ≥35 years in Tianjin^10^. However, differences in policy coverage and enforcement across regions limit a comprehensive national assessment. Among major cities, Shanghai ranks highest in tobacco control performance, including policy implementation and smoke-free environment creation^11^. Nevertheless, the specific health impacts of its comprehensive smokefree legislation remain insufficiently evaluated.

Shanghai has been at the forefront of local tobacco control in China. It introduced one of the earliest local smoking bans in 1994 and adopted more systematic regulations in 2010, following the implementation of the FCTC^12^. In 2017, Shanghai strengthened its legislation by tightening penalties and enforcing a comprehensive indoor smoking ban in all public places, workplaces, and public transportation, then further revised the regulations in 2022 to include e-cigarettes^13,14^. These stringent policies underscore Shanghai’s strong commitment to tobacco control, setting a standard for other cities in China. Since the implementation of smoke-free legislation, the adult smoking rate has decreased from 26.9% to 19.2%, achieving a consistent decline over 13 years and meeting the Healthy China 2030 tobacco control target in advance^15,16^. Moreover, Shanghai’s comprehensive smoke-free legislation has brought notable macroeconomic benefits. A recent study estimated that implementing similar policies nationwide could yield economic gains equivalent to 0.04–0.07% of GDP from 2017 to 2035, underscoring the broader value of tobacco control^17^. Despite significant achievements in enforcement, reduced smoking rates, and economic benefits, there is a lack of systematic evaluation on the health impacts, particularly in cardiovascular diseases such as ischemic heart disease (IHD). This gap needs to be addressed.

This study examines the impact of Shanghai’s comprehensive smoke-free legislation introduced in March 2017 on hospitalization and mortality rates of ischemic heart disease (IHD). We seek to provide evidence supporting policies to mitigate the cardiovascular disease burden in urban populations and offer recommendations for national and global tobacco control policies.

## METHODS

### Study design

This study adopted the Interrupted Time Series (ITS) method to analyze the effects of the intervention. ITS is commonly used in public health, public policy, and health services fields to evaluates the impact of an intervention, including changes in level and trend after the interruption^18^.

The intervention policy evaluated in this study was the revised Shanghai’s comprehensive public smoking ban, which came into effect on 1 March 2017. The primary health outcomes assessed were the monthly hospitalization rate and mortality rate of ischemic heart disease (IHD) among registered residents of Shanghai. In addition to the policy, the study considered covariates known to influence the risk of ischemic heart disease over time based on biological plausibility and prior epidemiological evidence, including seasonal effects, meteorological conditions, and air pollution index^19,20^.

### Data collection

The study population included all IHD-related hospitalizations and deaths among registered residents of Shanghai. The hospitalization data were obtained from the Shanghai Municipal Health Commission for the period from July 2013 to December 2021. Hospitalized patients were identified based on the principal discharge diagnosis of IHD (I20-I25). To avoid double counting, readmissions for the same condition within 28 days were excluded, while readmissions after 28 days were considered as new cases.

Mortality data were obtained from the All-Cause Mortality Surveillance System maintained by the Shanghai Municipal Center for Disease Control and Prevention. The cause of death classification for the study period was based on 10th revision of the International Classification of Disease. The underlying causes of death selected for analysis included IHD (I20-I25).

Covariates such as meteorological conditions and air pollution data were also collected. Monthly average temperature (°C) and relative humidity (%) from July 2013 to December 2021 were sourced from the China Meteorological Data Sharing Service System. As PM2.5 monitoring data were unavailable from the Shanghai Environmental Monitoring Center prior to 2015, those were obtained from the Tracking Air Pollution (TAP) near real-time air pollutant concentration database, a method widely used in previous studies^21^. Population data for registered residents were obtained from the Shanghai Statistical Yearbook. To estimate the monthly registered population, the average of the year-end population from the preceding and subsequent year was calculated.

### Statistical analysis

Three methods were used to analyze the short-term and long-term effects of Shanghai’s comprehensive smoke-free legislation on the monthly IHD hospitalization and mortality rates among registered residents.

Firstly, an interrupted time series analysis was conducted based on the autoregressive moving average (ARMA) model and OLS regression models adjusted for autocorrelation^22^. Given the small sample size of IHD hospitalizations and deaths among residents aged <35 years, this study limited the analysis to residents aged ≥35 years. The primary outcome variables were the monthly hospitalization and mortality rates of IHD (per 100000 people), calculated by dividing the monthly number of hospitalizations or deaths by the estimated monthly population. A dummy variable was used to define the policy intervention, with a value of 0 for the pre-intervention period and 1 for the postintervention period. An interaction term between the policy and time was also included to estimate changes in the post-trend slope after policy implementation compared to the pre-period. The policy intervention point was set at 1 March 2017, and the study period spanned between 1 July 2013 and 31 December 2021 (102 months in total: 44 months pre-intervention and 58 months post-intervention). The ITS regression model used was as follows:

Y = β_0_ + β_1_Tt + β_2_Xt + β_3_XtTt + ε_t_

where Y represents the outcome variable (monthly hospitalization or mortality rate); Tt represents the time since the start of the study; Xt is a dummy variable representing the intervention (0 for preintervention, 1 for post-intervention); and XtTt is an interaction term; β_0_ is baseline hospitalization or mortality rate at the start of the study; β_1_ is the slope of the outcome variable before intervention introduced; β_2_ represents the immediate level change in the outcome variable after the intervention and β_3_ represents the difference in trend slopes before and after the intervention.

Secondly, to verify the policy’s impact on different subgroups, stratified analyses by age (35–64 years and ≥65 years) were conducted. Given the United Nations’ threshold for aging, aged 65 years was used to define age groups^23^. However, due to China’s aging population trend from 2013 to 2021 and the variability in population size across age groups, the age-standardized rate was performed to minimize bias from comparing crude rates^24,25^. In this article, we define the registered population of Shanghai in 2013 as the standard population, adjusting hospitalization and mortality rates for the years 2014–2021 to reflect the policy’s true impact on different age groups.

Lastly, ITS analyses were performed using negative binomial and Poisson distribution models in the sensitivity analysis. The outcome variable was the monthly number of IHD hospitalizations or deaths, with the annual population set as an offset variable (fixed coefficient=1) to estimate the rates. The models incorporated dummy variables for the intervention and an interaction term for policy and time. In addition, seasonal variations were modeled using Fourier series terms, including annual sine and cosine functions^26^. Moreover, the model adjusted for monthly average temperature, relative humidity, and PM2.5 concentrations, which have been shown to be associated with cardiovascular events in previous studies^20^.

All statistical tests were two-tailed, and a p<0.05 was considered statistically significant. Analyses were performed using Stata V.13, with statistical modeling conducted using the *itsa* package and the *glm* function^27^.

### Patient and public involvement

The data used in this study were obtained from the Shanghai Municipal Health Commission and the Shanghai Municipal Center for Disease Control and Prevention. As the outcome measures were aggregated monthly data, patients and the public were not directly involved in the study design, data analysis, or interpretation of results.

## RESULTS

### Descriptive statistics

From 1 July 2013 to 31 December 2017, there were 898535 hospitalizations due to ischemic heart disease (IHD) and 180658 deaths caused by IHD among registered residents of Shanghai (Table 1).

**Table 1:** Annual hospitalization rates, mortality rates (per 100000), and adult smoking rates for IHD among registered residents aged ≥35 years in Shanghai (2013–2021)

*Year*	*Annual hospitalizations* *n*	*Hospitalization rate* *(year-to-year %* *change)* *n (%)*	*Annual deaths* *n*	*Mortality rate (year-to-* *year % change)* *n (%)*	*Adult smoking rate* *(year-to-year %* *change)* *% (%)*
2013	39158	816.63*	7874	164.21*	26.7
2014	85246	876.84 (7.37)	17524	180.25 (9.77)	25.6 (-4.12)
2015	91710	931.79 (6.27)	19057	193.62 (7.42)	23.3 (-8.98)
2016	103079	1033.07 (10.87)	19925	199.69 (3.14)	21.0 (-9.87)
2017	114238	1124.87 (8.89)	20366	200.54 (0.43)	20.2 (-3.81)
2018	121104	1173.02 (4.28)	22069	213.76 (6.59)	19.9 (-1.49)
2019	123702	1193.77 (1.77)	23504	226.82 (6.11)	19.7 (-1.01)
2020	105934	1001.39 (-16.12)	24150	228.29 (0.65)	19.4 (-1.52)
2021	114364	1067.85 (6.64)	26189	244.54 (7.12)	19.4 (0.00)

* Adjusted for annual hospitalization and mortality rates based on half-year data.

Both the annual hospitalization rate and mortality rate for IHD showed an overall upward trend during the study period; however, the upward trend slowed after the implementation of Shanghai’s comprehensive smoke-free legislation in 2017. Regarding the annual hospitalization rate, the increase began to slow in 2017 and continued to decelerate until 2020, when it reached its largest year-to-year decrease (-16.12%). Similarly, the annual mortality rate for IHD saw the smallest year-to-year increase in 2017 (0.43%). After a slight rise, it reached the second-lowest yearto-year growth in 2020 (0.65%). During the same period, the adult smoking rate showed a declining trend, decreasing from 26.7% to 19.4%. However, the decline slowed after 2017.

### Impact of Shanghai’s comprehensive smoke-free legislation on hospitalization rates of ischemic heart disease

**Figure 1:**
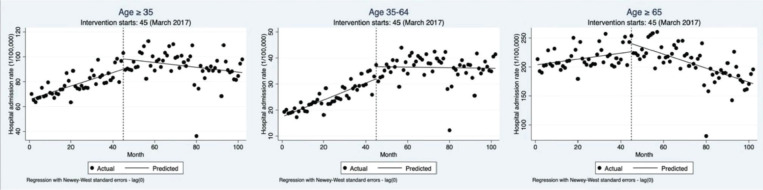
Monthly hospitalization rates of ischemic heart disease (IHD) among the registered population and age subgroups in Shanghai from July 2013 to December 2021 before and after the implementation of the comprehensive smoke-free legislation. The black circles represent the monthly hospitalization rate, the black line represents the time trend of hospitalization rates, and the vertical dashed line indicates the time of policy implementation

After the implementation of Shanghai’s comprehensive smoke-free legislation in March 2017, the monthly hospitalization rate for IHD among registered residents aged ≥35 years showed a significant step increase (β=8.29; 95% CI: 2.45–14.13). However, the long-term post trend showed a significant decline (β= -0.73; 95% CI: -0.93 – -0.54), preventing an estimated 890 hospitalizations annually for residents aged ≥35 years (Figure 1, Table 2).

**Table 2:** Baseline, age standardized, and sensitivity analysis results for the impact of Shanghai’s comprehensive smoke-free legislation on IHD hospitalization rates (age-stratified)

*Hospitalization* *rate*	*Baseline results*			*Age standardized*		*Adjusted for covariates (Poisson)*		*Adjusted for covariates* *(Nbinomial)*	
*IHD*	*Step-change* *(95% CI)*	*Post-trend* *(95% CI)*	*Hospitalizations* *avoided* *annually*	*Step-change* *(95% CI)*	*Post-trend* *(95% CI)*	*RR* *(95% CI)*	*RR* *(95% CI)*	*RR* *(95% CI)*	*RR* *(95 % CI)*
Total (≥35)	8.29* (2.45–14.13)	-0.73* (-0.93 – -0.54)	890	8.29* (2.45–14.13)	-0.73* (-0.93 – -0.54)	1.11* (1.10–1.12)	0.99* (0.99–0.99)	1.11 (0.22–1.99)	0.99 (0.96–1.02)
Subgroup (35–64)	4.68* (2.11–7.26)	-0.33* (-0.42 – -0.25)	280	3.46* (1.56–5.37)	-0.24* (-0.31 – -0.18)	1.13* (1.12–1.15)	0.99* (0.99–0.99)	1.14 (0.23–2.04)	0.99 (0.96–1.01)
Subgroup (≥65)	13.96 (-0.67–28.58)	-1.72* (-2.21 – -1.24)	636	3.65 (-0.18–7.47)	-0.45* (-0.58 – -0.32)	1.10* (1.08–1.11)	0.99* (0.99–0.99)	1.09 (0.22–1.97)	0.99 (0.96–1.02)

Covariates include monthly average temperature, relative humidity, and PM2.5 concentrations. RR: risk ratio.

* p<0.05.

Age subgroup analysis revealed that among residents aged 35–64 years, the hospitalization rate significantly increased after policy intervention (β=4.68; 95% CI: 2.11–7.26), but the long-term post trend showed a significant decline (β= -0.33; 95% CI: -0.42 – -0.25). Compared to the age group of 35–64 years, the step increase in hospitalization rates for residents aged ≥65 years was larger but not statistically significant (β=13.96; 95% CI: -0.67– 28.58). However, the long-term decline in this group was more significant (β= -1.72; 95% CI: -2.21 – -1.24) (Figure 1, Table 2). Additionally, standardizing age structure using the 2013 registered population showed that the standardized hospitalization rate trends were consistent with the baseline results for all age groups (Table 2).

In sensitivity analyses, which controlled for seasonal trends and meteorological confounders, the overall trends of Poisson and Nbinomial models’ results were consistent with the baseline findings (Table 2). In the Poisson model, for residents aged over 35 years, the hospitalization rate significantly increased immediately after the intervention (RR=1.11; 95% CI: 1.10–1.12) and showed a significant post-trend decline (RR=0.99; 95% CI: 0.99–0.99). Also, the trend and significance for the two age subgroups were consistent with the overall population. While in the Nbinomial model, the overall hospitalization rate showed a step increase (RR=1.11; 95% CI: 0.22– 1.99) and a post-trend decline (RR=0.99; 95% CI: 0.96–1.02), but neither were statistically significant. Results for the age subgroups were consistent with the overall population.

### Impact of Shanghai’s comprehensive smoke-free legislation on mortality rates of ischemic heart disease

**Figure 2:**
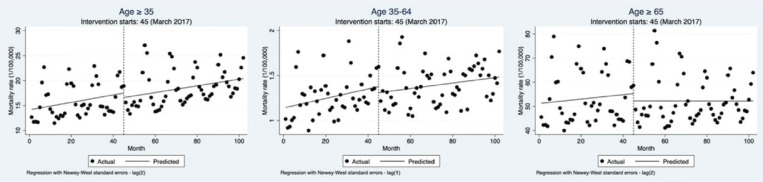
Monthly mortality rates of IHD among the registered population and age subgroups in Shanghai from July 2013 to December 2021 before and after the implementation of the comprehensive smoke-free legislation. The black circles represent the monthly mortality rate, the black line represents the time trend of mortality rates, and the vertical dashed line indicates the time of policy implementation

The results for monthly mortality rates differed from the hospitalization rates, as there was no evidence that the policy intervention led to a significant reduction in mortality rates. Among residents aged >35 years, the mortality rate showed a slight decline on step change (β= -0.90; 95% CI: -4.76–2.95) and an insignificant decrease on post trend (β= -0.0075; 95% CI: -0.14– 0.12) (Figure 2, Table 3).

**Table 3:** Baseline, age standardized, and sensitivity analysis results for the impact of Shanghai’s comprehensive smoke-free legislation on IHD mortality rates (age-stratified)

*Mortality rate*	*Baseline results*		*Age standardized*		*Adjusted for covariates (Poisson)*		*Adjusted for covariates (Nbinomial)*	
*IHD*	*Step-change* *(95% CI)*	*Post-trend* *(95% CI)*	*Step-change* *(95% CI)*	*Post-trend* *(95% CI)*	*RR* *(95% CI)*	*RR* *(95% CI)*	*RR* *(95% CI)*	*RR* *(95 % CI)*
Total (≥35)	-0.90 (-4.76–2.95)	-0.0075 (-0.14–0.12)	-0.90 (-4.76–2.95)	-0.0075 (-0.14–0.12)	0.99 (0.98–1.02)	0.99 (0.99–1.00)	0.99 (0.20–1.78)	0.99 (0.97–1.03)
Subgroup (35-64)	-0.068 (-0.26–0.13)	-0.0022 (-0.01–0.00)	-0.05 (-0.19–0.09)	-0.0016 (-0.01–0.00)	0.99 (0.90–1.07)	0.99 (0.99–1.00)	0.97 (0.20–1.75)	0.99 (0.97–1.03)
Subgroup (≥65)	-2.99 (-15.08–9.09)	-0.093 (-0.52–0.33)	-0.78 (-3.93–2.37)	-0.024 (-0.13–0.09)	1.00 (0.98–1.02)	0.99* (0.99–0.99)	0.99 (0.20–1.78)	0.99 (0.97–1.03)

Covariates include monthly average temperature, relative humidity, and PM2.5 concentrations. RR: risk ratio.

* p<0.05.

Age subgroup analysis results were consistent with the overall population, which were also not statistically significant. For residents aged 35–64 years, mortality rates showed a slight short-term decline (β= -0.068; 95% CI: -0.26–0.13), and the post-trend downward trend was not significant (β= -0.0022; 95% CI: -0.01– 0.00). For residents aged ≥65 years, the step change (β= -2.99; 95% CI: -15.08–9.09) and post trend declines (β= -0.093; 95% CI: -0.52–0.33) were larger than for the age group of 35–64 years but remained insignificant (Figure 2, Table 3). Standardized mortality rate results were consistent with baseline results for all age groups (Table 3).

In sensitivity analyses, the results of the Nbinomial model were consistent with baseline findings, showing slight but insignificant short-term and long-term declines in mortality rates (Table 3). In the Poisson model, the mortality results for the age group of ≥35 years and 35–64 years were consistent with baseline results. However, among residents aged ≥65 years, mortality rates slightly increased immediately (RR=1.00; 95% CI: 0.98–1.02) and significantly declined post the intervention (RR=0.99; 95% CI: 0.99–0.99).

## DISCUSSION

This study used interrupted time series analysis with multiple models to evaluate the impact of the comprehensive smoke-free legislation in Shanghai, implemented on March 2017, on the hospitalization and mortality rates of ischemic heart disease (IHD) in the local resident population aged ≥35 years. Between 1 July 2013 and 31 December 2021, we observed a long-term decline in hospitalization rates for ischemic heart disease that may be related to the implementation of Shanghai’s smoke-free policy, while no statistically significant change was found in mortality rates.

At baseline, the hospitalization rates of IHD for registered residents aged ≥35 years in Shanghai showed an immediate significant increase after the comprehensive public smoking ban, followed by a significant post-trend decline. This trend of significant long-term decline rather than immediate decline is consistent with the results of a study on acute myocardial infarction in Qingdao^28^. Research in Chile, Germany, New York, Singapore and Switzerland also reported reductions in annual cardiovascular disease hospitalizations after the implementation of tobacco control policies^6,29-32^. In contrast, The Regulations of Shanghai Municipality on Smoking Control in Public Places are officially amended and took effect in March 2017, but actually the regulations had been adopted at the 33rd Session of the Standing Committee of the 14th Shanghai Municipal People’s Congress on 11 November 2016^13^. Therefore, the public’s adherence was weaker when undergoing a transitional period prior to implementation. Moreover, some smokers may have continued their habits in certain nonsmoking areas (such as at home), leading to increased exposure to secondhand smoke, particularly affecting vulnerable groups like the elderly and resulting in short-term negative health impacts^33^. Additionally, this period coincided with winter, which is a highincidence season for ischemic heart disease and other cardiovascular conditions^19^. This seasonal effect could have further contributed to the short-term increase in hospitalization rates. Furthermore, the ‘Regulations’ clearly required Municipal Health Promotion Commission, relevant administrative departments and mass media to carry out publicity and education on smoking control, particularly regarding the risks of smoking and cardiovascular disease^13^. Those efforts may raise public awareness and lead more people to seek medical help immediately when symptoms such as chest pain appeared, which likely contributed to the short-term rise in IHD hospitalizations. Interestingly, a study in Xi’an showed an immediate 31.66% decrease in IHD hospitalizations following smoking control policy intervention, with a subsequent significant 40.58% increase in annual hospitalizations^34^, which contrasts with the trend observed in our study. The difference in long-term trends highlights Shanghai’s superior enforcement and intervention effectiveness because of strict penalties for smoking violations (personal: Shanghai vs Xi’an: 50–200 vs 10; venue: Shanghai vs Xi’an: 2000–10000 vs 500–1000)^35^. Since the comprehensive public smoking ban was implemented in 2017, the annual average fines for tobacco control enforcement have exceeded 2.2 million Chinese Yuan, with a cumulative total nearing 16 million Yuan by the end of 2023. This penalty amount ranks first among all 31 provinces in China. Furthermore, 88.7% of Shanghai residents are aware of the regulations, and the support rate for the public smoking ban reaches 98%^36^. This widespread awareness and strong public support ensure the effectiveness of the policy, leading to long-term health benefits.

For IHD mortality rates among residents aged ≥35 years, the study found minor declines both immediately and over the long-term, but these were not statistically significant. Similar findings were reported in Qingdao, where no significant effects on acute myocardial infarction mortality rates were detected post-intervention^28^. Shanghai is a pioneer in tobacco control legislation in China, making a significant decline in smoking rates in the early years (a 6.9% decrease from 2013 to 2018). However, the rate of decline has slowed in recent years, possibly entering a ‘diminishing returns’ phase. Nonetheless, Shanghai continues to progress steadily toward the target of reducing the adult smoking rate to 18% as outlined in the ‘Healthy Shanghai Action (2019– 2030)’^37^. Additionally, Shanghai has abundant medical resources, with cardiovascular disease diagnosis and treatment among the best in China. Since 2016, the establishment of pre-hospital treatment systems for chest pain patients, together with advancements in medical technology, has improved the management of ischemic heart disease^38^. These medical improvements may extend the survival of patients with ischemic heart disease, resulting in a delayed and gradual reduction in mortality rates which means a ‘lag effect’. Finally, cardiovascular diseases are also influenced by comorbidities such as diabetes and hypertension^39^. Research shows that the prevalence of hypertension and diabetes among Shanghai residents aged 35– 74 years has increased significantly from 2002 to 2017, which may offset some of the health benefits of tobacco control policies^40^. As a result, the slow recent decline in adult smoking rates, improvements in treatment technologies, and rising comorbidity rates may explain the lack of statistically significant reduction in the mortality rates of IHD.

To account for population aging, we analyzed age-standardized hospitalization and mortality rates and observed consistent results with the baseline findings. Notably, the intervention’s effects were more pronounced among individuals aged ≥65 years, aligning with results from Chile and Hong Kong^6,41^. In Hong Kong, the IHD mortality rate among individuals aged ≥65 years decreased by 9.9%, while a slight increase was observed in younger populations. This may be because elderly people in Hong Kong often appear in restaurants, tea houses and mahjong parlors, resulting in a relatively higher exposure to secondhand smoke^41^. Shanghai’s comprehensive public smoking ban amended in 2017 mandates smoking shall be prohibited in indoor public places and public transport vehicles, including waiting areas for public transportation where crowds occur^13^. These interventions in areas frequented by the elderly may be the reason we observed an important impact of smoke-free legislation on the hospitalization and mortality rates from ischemic heart disease among individuals aged ≥65 years.

Sensitivity analyses using Poisson and negative binomial models confirmed the robustness of the results, with consistent trends across models. Although the negative binomial model fit better due to lower AIC and BIC values (Supplementary file), its results were not statistically significant. This unusual difference has also been found in other studies, probably because Poisson regression makes more assumptions about the underlying data structure than negative binomial regression^42^. In this study, the data distribution is relatively concentrated, with a small degree of dispersion, and the changes before and after the policy are relatively stable. Therefore, Poisson model can better fit the fluctuations, and related international studies are also mainly based on the Poisson distribution^43,44^. This study uses two models to simultaneously verify the main trend results, which further improves the reliability of the research.

### Strengths and limitations

This study has several strengths. First, our data were sourced from the Shanghai Municipal Health Commission and the all-cause mortality surveillance system, covering a large and relatively closed population, which reduces selection bias and enhances internal validity, thus improving the reliability and generalizability of the results. Additionally, the data span a long period (102 months in total, with 44 months prior to the policy), which effectively captures the long-term effects of the policy intervention while reducing the influence of random events and improving model fit. Second, we used standardized hospitalization and mortality rates to reflect the effects of the policy intervention, eliminating the impact of aging trends, thus providing stronger and more objective support for the study’s findings. Furthermore, we used multiple models to control for covariates such as seasonality, weather conditions, and air pollution, ensuring the accuracy and robustness of the results. Finally, we presented changes in adult smoking rate as an important indicator directly affected by the smoking control policy, linking them with health outcomes to provide more solid evidences.

However, several limitations should be acknowledged. First, we did not include a control group of diseases unrelated to smoking, as their hospitalization and mortality trends significantly differed from those of ischemic heart disease (IHD) prior to the intervention. This lack of comparability precluded the use of a multi-group interrupted time series design and necessitated a single-group design, which may yield different results compared to studies with external or synthetic control groups – a known limitation of single-group interrupted time series analyses^45^. Future research could incorporate data on multiple smokingunrelated diseases and apply an iterative selection process to identify appropriate control groups, thereby enhancing the credibility of the results. Second, although we conducted age-stratified analyses, we were unable to perform sex-specific subgroup analyses due to data constraints. Given the substantial gender gap in smoking prevalence in Shanghai in 2021 (36.8% in males vs 0.8% in females), future studies should aim to incorporate sex-disaggregated data to assess differential policy impacts by gender^16^. Third, although we adjusted for several important time-varying environmental covariates, we could not control for individual-level covariates such as smoking status, socioeconomic status, cigarette affordability, and comorbidities due to the ecological nature of the study. This raises the possibility of residual confounding and limits causal interpretation. Fourth, the generalizability of our findings may be limited to settings with similar smoking prevalence patterns, policy environments, and health system contexts. Extrapolation to other countries or regions should be approached with caution. Finally, we treated IHD as a single aggregated outcome, despite potential heterogeneity across cardiac phenotypes, such as myocardial infarction, angina and sudden cardiac death. Future analyses could explore more granular outcome definitions to assess whether policy effects vary across specific subtypes of IHD.

## CONCLUSIONS

This study assessed the impact of the Regulation on Smoking Control of Shanghai amended in 2017, on hospitalization and mortality rates for ischemic heart disease (IHD) in the local resident population. We found that the comprehensive public smoking ban appears to be associated with a downward trend in the monthly IHD hospitalization rates, particularly among the population aged ≥65 years, but no significant association was observed with reduction in IHD mortality rates. This study provides reliable data to support the health benefits of comprehensive public smoking ban and confirms the preventive value for ischemic heart disease and other cardiovascular diseases. Future efforts should focus on strengthening the supervision and optimization of policy implementation to achieve greater public health benefits.

## Data Availability

The data supporting this research are available from the authors on reasonable request. Hospital admissions data for Shanghai registered population were obtained from the Shanghai Municipal Health Commission Information Center. Mortality data were obtained from the Shanghai Center for Disease Control and Prevention. Monthly meteorological data were obtained from the Shanghai Meteorological Bureau (http://sh.cma.gov.cn/sh/tqyb/wrtqyb/). PM2.5 concentration data were retrieved from a near-real-time air pollutant concentration database (http://tapdata.org.cn/). Data on the registered population were derived from the Shanghai Statistical Yearbook (https://tjj.sh.gov.cn/tjnj/index.html).
